# Quantitative elemental imaging in eukaryotic algae

**DOI:** 10.1093/mtomcs/mfad025

**Published:** 2023-04-25

**Authors:** Stefan Schmollinger, Si Chen, Sabeeha S Merchant

**Affiliations:** California Institute for Quantitative Biosciences (QB3), University of California, Berkeley, CA 94720, USA; Departments of Molecular and Cell Biology and Plant and Microbial Biology, University of California, Berkeley, CA 94720, USA; X-ray Science Division, Argonne National Laboratory, Lemont, IL 60439, USA; California Institute for Quantitative Biosciences (QB3), University of California, Berkeley, CA 94720, USA; Departments of Molecular and Cell Biology and Plant and Microbial Biology, University of California, Berkeley, CA 94720, USA

**Keywords:** heavy metal detoxification, iron, copper, XRF, SXRF, Chlamydomonas

## Abstract

All organisms, fundamentally, are made from the same raw material, namely the elements of the periodic table. Biochemical diversity is achieved by how these elements are utilized, for what purpose, and in which physical location. Determining elemental distributions, especially those of trace elements that facilitate metabolism as cofactors in the active centers of essential enzymes, can determine the state of metabolism, the nutritional status, or the developmental stage of an organism. Photosynthetic eukaryotes, especially algae, are excellent subjects for quantitative analysis of elemental distribution. These microbes utilize unique metabolic pathways that require various trace nutrients at their core to enable their operation. Photosynthetic microbes also have important environmental roles as primary producers in habitats with limited nutrient supplies or toxin contaminations. Accordingly, photosynthetic eukaryotes are of great interest for biotechnological exploitation, carbon sequestration, and bioremediation, with many of the applications involving various trace elements and consequently affecting their quota and intracellular distribution. A number of diverse applications were developed for elemental imaging, allowing subcellular resolution, with X-ray fluorescence microscopy (XFM, XRF) being at the forefront, enabling quantitative descriptions of intact cells in a non-destructive method. This Tutorial Review summarizes the workflow of a quantitative, single-cell elemental distribution analysis of a eukaryotic alga using XFM.

## Graphical Abstract

**Graphical Abstract:**
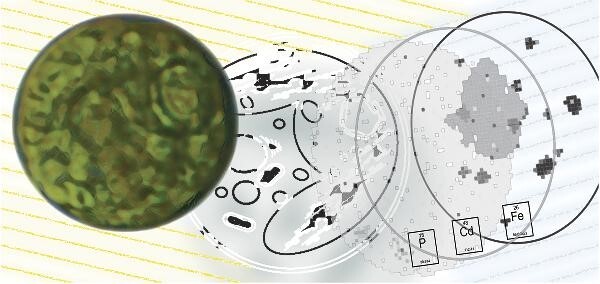
Elemental imaging of a single-celled eukaryotic alga using X-ray fluorescence.

The aim of this review is to (1) highlight the contributions of different elements to photosynthetic life and the concepts of how organisms control their elemental composition, (2) introduce the methodologies involved in studying elemental distributions in cells, especially XFM, (3) review the current state of XFM studies in eukaryotic algae, and (4) extract a methodology framework for conducting XFM studies from these works. It is our goal to facilitate the entry into the field of elemental research for algae scholars encountering questions of metal homeostasis and elemental heterogeneity for the first time, and to encourage the use of quantitative elemental imaging approaches for the purpose of determining biological function.

## Elemental composition of cells

**Fig. 1:**
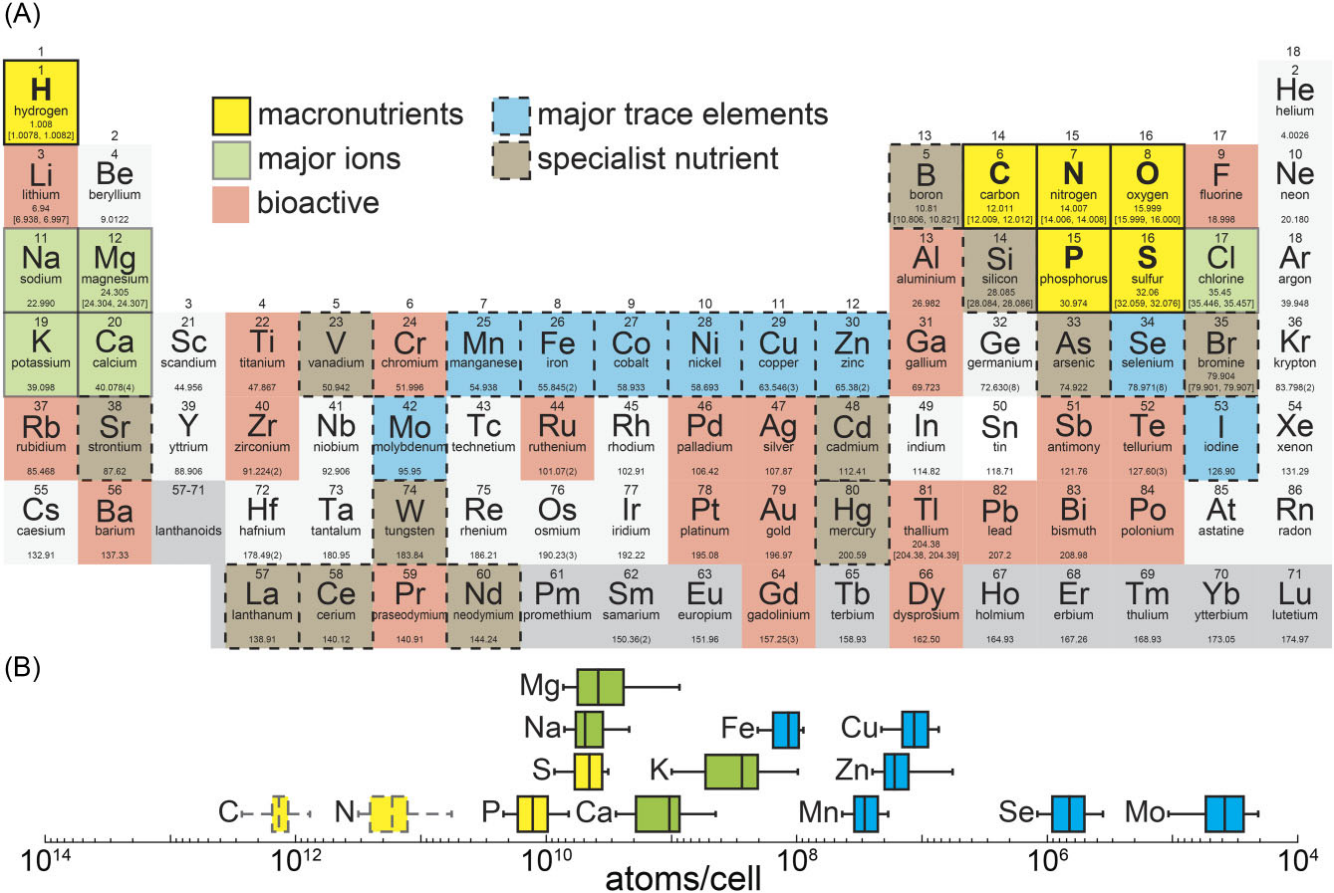
Elements of interest in life. (A) Periodic table of the elements showing relevant elements to living organisms. Elements are grouped into macronutrients (CHNOPS, yellow background), the major cations (Na, Mg, K, and Ca, green background), major trace elements relevant for enzymatic function in many organisms (blue background), nutrients that are relevant for specialist's enzyme function (brown background), and elements that affect metabolism (beneficial or toxic) in living organism without being directly utilized in enzymes (bioactive, red background). (B) Elemental composition of the eukaryotic green algae *C. reinhardtii*. Solid lines indicate ICP-MS/MS analysis; dotted lines indicate TOC/TN measurements.

The elements of the periodic table are the indivisible foundation of all matter, including all biological life on our planet (Fig. 1). Every component of a cell is assembled from a careful selection of elements, which can either be essential to the organism, defined by their irreplaceability and absolute requirement to complete the vegetative or reproductive life cycle,^1^ or provide specific beneficial advantages, improving the organism’s fitness in general or in specific situations or environmental niches. The most prominent elements in living matter are C, H, N, O, P, and S, which are essential, account for most of biomass, and constitute the backbone of proteins, carbohydrates, nucleic acids, and lipids.^2–4^ In addition, there are many other elements that are useful to living organisms in lower abundance.^1^ For example, it is estimated that ∼40% of all enzymes utilize a uniquely suited element outside the group of macronutrients (CHNOPS) within their catalytic centers to enable catalysis.^5,6^ The range of essential or beneficial elements can change between different organisms depending on the organism's environmental niche and its metabolism, the latter consequently defining the organism's enzyme portfolio.

Most organisms use cations (K,^7–10^ Mg,^11–15^ Ca,^16–19^ and to a lesser degree Na^20–24^) and the Cl anion,^25–28^ all of which are abundant constituents of biological matter, to regulate osmotic pressure and pH, build gradients across membranes that facilitate energy production, transport processes, or signal transduction.^29^ These ions also serve as allosteric regulators of enzyme activity,^9,30^ even participate in catalysis (e.g. Mg in isocitrate lyase^31,32^ or Ca in the oxygen-evolving complex of PSII^33^), or bind to metabolites (most importantly, Mg-ATP and Mg-chlorophyll), critically enabling their utility.

Additionally, organisms require various additional sets of elements in trace amounts (including many metal ions) to enable critical chemical functionalities that are not provided from functional groups found on metabolites or amino acids.^4,34^ These micronutrients/trace elements are often required co-factors for metabolically essential enzymes, and consequently, their acquisition, intracellular distribution, and utilization are crucial aspects of cellular metabolism.^4^ Trace elements typically present the most interesting targets for elemental imaging because of the impact of their chemistry on cell health and metabolism, the range of their abundances in an organism, and the dynamic regulation involved in their utilization.^4,35^

A set of trace elements is essential for most organisms, including Fe,^36–38^ Cu,^39–43^ Co,^44–47^ Ni,^48–51^ Mn,^52–55^ Zn,^56–60^ Se,^61^–^70^ I,^71–74^ and Mo.^75–77^ Many of these elements have oxidation states that are stable under physiological conditions, and a role in redox chemistry is a common function for many of the trace elements. Fe, Cu, and Mn, with multiple stable oxidation states, are therefore among the most abundant and important trace elements for all organisms; at lower abundance, Ni, Co, and Mo are also required by many organisms.^5,36,39,44,48,52,56,75,78–80^ Fe is used most famously in hemoglobin for O_2_ transport, but has many other uses, e.g. in electron transport (e.g. in complex I, II, and III of the mitochondrial electron transfer chain) and as an essential catalyst in many critical enzymes (e.g. in aconitases, catalases, and nucleases), either bound directly to amino acids or alpha-ketoglutarate, complexed within the tetrapyrrole heme, or assembled into Fe–S clusters.^81–86^ Similarly, Cu can also be used for both oxygen (e.g. hemocyanin) or electron transport (e.g. cytochrome *c* oxidase), and can directly be bound to amino acids in catalytic centers of enzymes^43,87,89–91^ Mn is also essential and critically involved in several enzymes in DNA metabolism, detoxification of reactive oxygen species, and carbohydrate metabolism.^92–95^ Fe, Cu, and Mn, in addition to their many important contributions to general metabolism, are also critically employed in photosynthetic electron transfer and chloroplast metabolism.^96,97^ Ni is used in enzymes in a wide range of organisms, e.g. in ureases and hydrogenases.^98^ Co is utilized as a cofactor in a few enzymes directly, but is most commonly utilized at the center of cobalamin (also known as vitamin B_12_), which is critical in methionine biosynthesis and nitrogen fixation.^44,99,100^ Mo, outside of bacterial FeMo-nitrogenase, is usually bound to the pterin cofactor Moco, which is used in many important enzymes, e.g. nitrate reductase, xanthine oxidoreductase, and sulfite oxidases.^75,101–103^ Zn is similarly abundant and widespread as a trace element as Fe, Cu, and Mn, but is used as a Lewis acid and a structural component for proteins in most organisms.^104–106^ Se, most prominently, is utilized as selenocysteine in specific enzymes requiring the element in their catalytic centers, e.g. glutathione peroxidases.^61,67,107–111^ I is used in thyroid hormones, essential for vertebrates, and has a role in oxidative stress response in algae.^71–74^

Other elements, V,^67–70,110,111^ B,^112–117^ Si,^118–121^ As,^122–124^ Br,^125–127^ Sr,^128–130^ Cd,^131–134^ W,^135–139,140^ Hg,^141–143^ La,^144–147^ Ce,^148–150^ and Nd,^151,152^ are not commonly used in most organisms, but some specialists have found unique roles for these elements.^144,146,148,151^ Additionally, many of these nutrients only useful to specialist are toxic, and other, bioactive elements for which no direct enzymatic use has yet been identified (Fig. 1), can accumulate in organisms involuntarily, hijacking uptake routes for other, similar ions. They interfere with biological processes, most commonly in detrimental fashion. Together with these harmful activities associated with the redox-active trace elements (Fe, Cu, Mn, Ni, Co, and Mo), trace nutrients pose an inherent risk to cell integrity. Therefore, the chemical reactivities that make these elements useful in the first place must be controlled intracellularly to avoid unintended reactions, e.g. by using compartmentalization or detoxification mechanisms.^153–155^ The concentration of many of these elements in the direct environment of a cell is a critical parameter, determining if the organism is starving for the element as a nutrient, when either abundance or bioavailability is low, or if cell health is threatened by overexposure, exceeding demand, and the capacity for detoxification/containment.^156^ The toxicity can either be directly attributed to detrimental reactivities of the element when uncontrolled, the production of secondary toxic products, e.g. reactive oxygen species, or enzyme/metabolite mis-metalation. Mis-metalation is largely attributed to the inherent flexibility in proteins and the similar physical properties (ionic radii, charge, and coordination preferences) of the biologically common trace metal.^157,158^ Most enzymes are tuned to function with a specific metal cofactor. Binding of a different, similar metal at the active site can result in loss-of-function, or worse, the production of unintended products or promotion of side-reactions.^154,159,160^ All organisms therefore carefully control their elemental composition at the point of uptake, resulting in specific cellular quotas, especially in the case of redox active trace elements. Cells also employ elaborate strategies to avoid mis-metalation intracellularly, including the compartmentalization of specific elements to ensure that the correct metal binds to newly synthesized proteins, or the use of metallochaperones to ensure correct delivery through protein–protein interactions.^161–163^ Some metals are associated with organic groups or build into large clusters (e.g. Fe in heme and Fe–S clusters) for similar reasons.

The pathways for trace element metabolism are among the most ancient in biology,^164^ and the general concepts involved in trace metal utilization are well conserved across organisms. Photosynthetic organisms specifically have unique requirements with respect to the elemental composition because of the metabolic demand of the photosynthetic apparatus and the specific pressures they experience from the environmental niches they occupy. Processes realized by abundant enzymes weigh harder on the specific elemental quotas, and proteins involved in photosynthetic carbon fixation (most prominently photosystems I and II, the Cyt *b*_6_*f* complex, and the enzymes of the Calvin–Benson cycle) are among the most abundant proteins in photosynthetic organisms. Magnesium is especially important here, with 25% of Mg in plants being found in chloroplasts, where it is integral for the light-capturing chlorophylls (Mg-containing tetrapyrroles) and in the activation of the carbon-capturing RuBisCO.^11,165,166^ The photosynthetic electron transfer chain also requires large amounts of Fe; 80–90% of Fe in leaves is found in the chloroplast.^167^ Most abundantly, Fe is found in photosystem I, which is utilizing three 4Fe–4S centers to transport electrons from the acceptor to the donor side, and in ferredoxin (2Fe–2S center), the soluble carrier protein distributing photosynthetically derived electrons to various chloroplast processes, including NADPH production, nitrogen assimilation, and chlorophyll biosynthesis.^97,168^ Cu in plastocyanin, the soluble electron carrier between the Cyt *b*_6_*f* complex and photosystem I, and Mn and Ca, in the oxygen-evolving complex of photosystem II, are also vital in photosynthetic electron transport.^17,39,169^ Zn is involved in photosynthetic CO_2_ fixation, in the catalytic center of carbonic anhydrases (CAHs), and in the assembly of RuBisCo.^170,171^

### Intracellular elemental heterogeneity

The elemental composition of an organism is a dynamic function of the metabolic needs of a cell. As mentioned above, it varies between organisms depending on the functions they employ, but even within a given species, it is adjusted between different metabolic or developmental stages, tissues, and cell types, and in response to the availability of nutrients and other stimuli in the environment.

Nutrient limitation is a major driver of acclimation responses to the elemental composition. While limitation to essential elements often results in cell cycle arrest or even cell death, the elemental composition of non-essential, but beneficial trace nutrients can be most variable. Fe bioavailability is low both in aquatic and cropland environments, mainly because of its low solubility in the more oxidized, but most prevalent, Fe(III) state.^172,173^ Its central role in the abundant photosynthetic apparatus, which most primary producers depend on for carbon fixation, assures that Fe availability limits virtually all forms of life.^174,175^ An evolutionary adaptation in some photosynthetic organisms to low Fe environments was therefore to use a Cu-containing protein, plastocyanin, instead of the Fe-containing Cyt *c*_6_, for the same function, namely, the transport of electrons between Cyt *b*_6_*f* and photosystem 1.^169,176^ While this reduces the organisms’ Fe quota, simultaneously, its dependence on Cu becomes greater. Other acclimation mechanisms also involve the intracellular recycling of metal cofactors and subsequent redistribution to other processes, according to a hierarchy of essentiality in the organism.^177–180^

Intracellular over-accumulation of various elements beyond the necessary quota can also affect cell health and occurs either in polluted or otherwise nutrient-imbalanced environments. Plants, e.g. accumulate P when Zn is limiting and vice versa,^181–183^ while the green alga Chlamydomonas accumulates large amounts of Cu in Zn-limiting conditions.^184,185^ Sequestration in specialized, intracellular compartments is a common strategy to either detoxify over-accumulating, biologically undesired elements, store scarce nutrients in preparation for periods of limitation or for future generations, sequester a resource away from competitors, or buffer nutrients temporarily during metabolic transitions.^186–189^ Acidocalcisomes are lysosome-related organelles, first identified in trypanosomes but widely present in eukaryotes.^190^ Acidocalcisomes are rich in Ca and P,^191,192^ in some organisms also K,^193^ and can temporarily accumulate various micronutrient metals and toxic elements. The eukaryotic green alga Chlamydomonas, e.g. has been found to sequester the trace nutrients Cu, Mn, and Fe and the heavy metal Cd in acidocalcisomes in periods of over-accumulation.^184,194–197^ Vacuoles in general are the most important storage sites in eukaryotes, including photosynthetic organisms,^187,189,198^ but other means of storage and sequestration can also be utilized. Proteins like ferritin, a soluble 24-subunit oligomer, can sequester trace elements, ∼4500 Fe ions in ferritins case,^199,200^ and other organelles like the starch-separated pyrenoid in the chloroplast have been found to contribute to Cd sequestration.^196,197,201^

Research of metal homeostasis not only aims to identify acclimation processes in natural settings but can also be exploited in bioremediation or biofortification applications. While heavy metals, *z* > 20, density >5 g/cm^3^,^202^ can be naturally present in specific soil or aquatic environments, more importantly, human activities, from mining and industrial production processes to domestic and agricultural practices, have led to an increased contamination of natural habitats with toxic metals.^203–205^ Cd, As, Pb, Cr, and Hg are thereby the most prominent elements, all presenting severe dangers to human health.^203^ While conventional methods like chemical precipitation, reverse osmosis, adsorption, or electrodeposition are used to remove heavy metals from environments, the use of biological organisms can be much more efficient and cost-effective.^206,207^ Photosynthetic algae, e.g. are uniquely positioned to be utilized in the removal of heavy metals in soil and aquatic environments, and research into their trace metal metabolism can greatly facilitate the effectiveness of these processes.^208–210^ Biofortification on the other hand, is a biotechnological process intended to increase the nutritional value of human nutrition, with major crop plants being generally poor sources of micronutrients.^211^ Targeting photosynthetic organisms like crops or algae, which are used as animal feed stocks or in natural supplements, is an efficient way to improve nutritional deficiencies.^212^

### Quantitative, intracellular elemental distributions

Whether it is for research or in the pursuit of biotechnological applications, the acquisition of spatially resolved, quantitative elemental maps is a key tool for researchers to identify the molecular mechanisms involved in elemental homeostasis. Elemental distribution can identify the function, specificity, and directionality of membrane transporters, which are involved in the uptake, removal, or intracellular distribution of specific nutrients in the cell. Transport mechanisms are equally involved in storage or sequestration efforts. Other proteins involved in elemental homeostasis, e.g. transcriptional/translational control factors, signal transduction components, chaperones, buffering/protective proteins or metabolites, and major client proteins, e.g. abundant enzymes requiring a specific elemental cofactor, are also required to achieve native elemental distribution. Using specific mutants and analyzing differential elemental distribution maps can facilitating the identification of the function of these proteins in the first place.^213^ Detailed elemental distribution maps in different stages of nutrition for individual elements or upon other environmental perturbations (pH, temperature, light) can help in identifying the molecular mechanisms utilized in acclimation.

Therefore, over the past decades, a number of analytical techniques have been developed to determine intracellular elemental distribution, utilizing different properties to distinguish the elements. The various techniques all have different advantages and disadvantages, making the different applications quite complementary, especially with regards to sensitivity (detection limit and quantifiable range), obtainable spatial resolution, range of elements that can be (simultaneously) recorded, sample preparation, preservation, the amount of material required, and the kind of artefacts produced either from sample preparation or from the methodology itself. For detailed reviews of the individual methodologies see,^214–216^ and an in-depth discussion of strengths and weaknesses between the most common individual techniques can be found here.^217^ Two major categories can be distinguished: (i) fluorescence microscopy-based techniques using element-sensitive dyes or genetically encoded metal-binding sensors and (ii) scanning technologies using either mass spectrometry [Laser Ablation Inductively Coupled Plasma Mass Spectrometry (LA-ICP-MS); nanoscale Secondary Ion Mass Spectrometry (nanoSIMS)] or the detection of element-specific energy signatures [Synchrotron Radiation X-Ray Fluorescence Microscopy, abbreviated either (XFM, XRF, SXRF, or SRXRF); Particle Induced X-ray Emission (PIXE); Energy Dispersive X-ray Spectroscopy, abbreviated either (EDX, EDS, or EDXS)] to determine the elemental composition.^214^

The fluorescence microscopy-based techniques rely on the chemical properties of the individual molecular probe to identify a specific element, sometimes even a specific oxidation state, resulting in a fluorescence change (reversible or irreversible). Coupled with an adequate fluorescence microscope with the necessary resolution, which is available in many laboratories, the probes allow one to quickly analyze intracellular elemental distributions in many cells.^218^ The analysis is limited to a set of compatible, non-overlapping fluorescent signals at a time, but the probes can be utilized in living cells to specifically assess the labile, accessible fraction of the element in the cell. Quantitation of the labile metal pool using fluorescence-based probes is possible,^219^ but access to particularly tightly bound, or less accessible, sequestered elemental cofactors might preclude capturing the total metal distribution using probes.

**Fig. 2:**
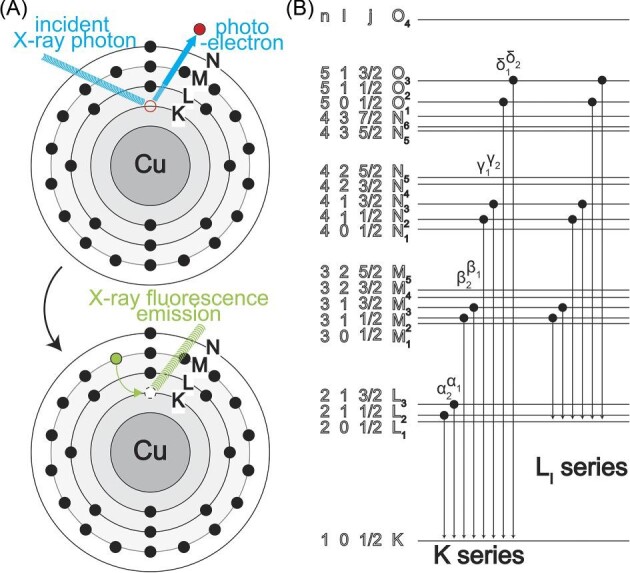
Principle of X-ray fluorescence emission. (A) Exemplary schematic of the physics at a Cu atom. An incident X-ray photon from a synchrotron source with sufficient energy to excite a core electron, leaving a hole in the process, which is subsequently filled by an electron from a different shell while emitting fluorescence equivalent to the energy difference. (B) Overview of the nomenclature of different electron transitions. For X-ray fluorescence microscopy, the K and L series are the most relevant for detection.

Outside of the probe-based techniques, XFM is a popular choice to determine quantitative intracellular elemental maps.^215,220^ XFM takes advantage of the unique electron orbital configurations of each element (Fig. 2). Using a highly focused X-ray beam at an energy above the binding energy of a core electron of an element can result in the removal of the electron from the atom. Upon removal, another electron from an outer shell can rapidly transition to the inner shell and fill the hole. The energy difference between the orbitals, specific for each transition (Fig. 2B), can be emitted as X-ray fluorescence, which can be recorded and analyzed using an energy-dispersive detector.^221^ Using a highly focused incident X-ray beam and a precisely controllable sample stage, 2D-projection images of the elemental composition of the material in the path of the beam are then assembled spot by spot at high resolution. At this point, most research utilizes highly brilliant synchrotron radiation sources to fast determine subcellular distribution maps at nanoscale, crucially limiting the amount of cells that can be analyzed.^216^ Benchtop systems, however, continue to improve in resolution^222^ and can present an attractive route to improve access to elemental imaging in the future. There are >50 synchrotrons globally, many of them offering XFM capabilities; pixel sizes below 100 nm can be achieved at multiple beamlines at synchrotrons including,^217,223–231^ which is well below the threshold of what is generally considered subcellular resolution (<1 μm, nanometer scale). Due to the ability of X-ray photons to penetrate biological material, XFM can be used to examine much thicker samples compared to those used for electron microscopy, often removing the requirement for sectioning, especially for single-cell organisms, and therefore facilitating sample processing and whole-cell analysis simultaneously.

Mass spectrometry-based techniques (LA‐ICP‐MS or nanoscale SIMS) are alternative options, offering isotope-specific detection of elements.^232^ In principle, these techniques use a highly focused laser (LA-ICP-MS) or ion beam (SIMS) to release material from a specific spot of a sample (cell section in the case of intracellular studies), which is then analyzed in a mass spectrometer for its elemental composition. Cs^+^ and O^−^ ion beams have a high ionization potential, allowing the determination of intracellular elemental maps with high detail, even of elements present in trace amounts.^184,215,232,233^ This has several advantages over XFM. By using the mass of an element instead of the electron configuration for detection, individual isotopes of element can be distinguished, allowing time-course studies with specific isotopes in pulse-chase mode. Lower resolution images of regions of the sectioned material allow to analyze a larger number of cells than can be analyzed with XFM. Both MS-based applications are considered destructive, in the sense that material of the specimen is used for the analysis, compared to the techniques that probe electron configurations or molecular probes, which are generally considered non-destructive, not consuming material for the identification of an element. The radiation, however, used for both of these techniques can, nevertheless, induce changes in the sample during detection. Radiation damage to cell structures, especially with long dwell times or after repeated scans, is a concern.^234^ While submicron resolution has recently been achieved (0.6 μm spot size^235^), the resolution of LA-ICP-MS is still one order of magnitude lower than the other techniques, limiting its utility to determine high-resolution intracellular structures in eukaryotic alga. SIMS at nanoscale can achieve similar resolutions to XFM, sub-100 nm pixel size, but matrix effects have long been a challenge for quantitative aspects. Additionally, the inherent nature of mass-spectrometry applications, which rely on the removal of material for elemental analysis, makes it difficult, but not impossible, to analyze whole cells quantitatively, requiring subsequent, repeated scans of the same area to fully capture the three-dimensional cell.^236^

### Eukaryotic algae as subjects

Eukaryotic algae are excellent subjects for quantitative elemental imaging studies. Algae is a term of convenience, combining several diverse eukaryotic groups of photosynthetic organisms.^237^ Many algae are unicellular eukaryotes of a size that is convenient for imaging applications; not too small to require too high of a resolution on the instrumentation end to determine subcellular distribution, but small enough to not require sectioning in applications that do not require it. They are important primary producers with crucial environmental roles, often inhabiting nutrient-limited environments on land or in the oceans, making them interesting research subjects. Green algae are within the Viridiplantae, which also contain the land plants. Together with the red algae (rhodophytes) and glaucophytes in the plantae supergroup, these algae are the result of primary endosymbiosis, where their chloroplasts arose from a free-living cyanobacterium.^238^ Outside the plant lineage, several diverse groups of algae are found: heterokonts (diatoms, brown algae), dinoflagellates, apicomplexans, haptophytes, crypotomonads, euglenoids, and chlorarachniophytes are all eukaryotic algae resulting from secondary or tertiary endosymbiosis, receiving their chloroplast from a eukaryotic donor.^237,239^ Within these groups, several organisms have been analyzed in detail, but probably no individual alga more than the green alga *Chlamydomonas reinhardtii*, and no group more than the diatoms of the genus Thalassiosira.

*Chlamydomonas reinhardtii* is a unicellular green alga that has been widely used as a eukaryotic, photosynthetic reference system. It is a haploid, facultative phototroph with a short generation time (∼6 h in the presence of a reduced carbon source). Its genome has been fully sequenced, and all three genomes can be targeted for modification.^240,241^ Chlamydomonas has been widely used for research on algal metabolism, and has served as a resource for commercial applications of algae as sources of biofuels or bioproducts.^242,243^ Its common ancestry with land plants, albeit distant (>1 billion years ago), allows for cross-comparisons to the intensively studied plant systems, and research performed in either to be cross-informative. Yet Chlamydomonas is less complex (single, uniform cell), contains smaller gene families and less complex gene structures. In terms of elemental composition, the photosynthetic electron transfer chain is virtually identical with that in land plants, and the alga utilizes a broad spectrum of metal cofactors to sustain its photosynthetic, respiratory, and metabolic capabilities. In the past decades, studies have been carried out to identify the major transporters involved in nutrient acquisition and distribution.^156,185,244–248^ Mechanisms for nutrient-sparing and recycling have been discovered,^169,177,249–251^ and storage sites were identified,^156,184,194,195,252–254^ making this organism particularly useful for elemental analysis studies.

Thalassiosira is a genus of centric diatoms found in diverse marine and freshwater ecosystems, widely recognized for their substantial contribution to global primary productivity.^255–257^ Their defining feature is the silicified cell walls, consisting of species-specific, fine-scaled nano-structures that are built intracellularly in a specialized compartment before being exported to assemble the cell wall.^258^ The genus contains mostly single-celled species, but, especially in marine species, the single cells can be connected via chitin fibrils to ‘string of beads’ colonies.^257^ The genomes of *Thalassiosira pseudonana*^259^ and *Thalassiosira oceanica*^260^ have been fully sequenced, and protocols for genetic manipulation of *T. pseudonana*^261^ and *Thalassiosira weissflogii*^262^ have been developed. Thalassiosira's elemental metabolism has been of particular interest to researchers, especially that of silicon, with regards to the synthesis of its cell wall, and iron, with regards to iron’s role in limiting the growth of alga in oceanic, high nutrient/low chlorophyll environments, restricting their potential for carbon sequestration.^260,263–266^ Algae have been excellent subjects for elemental imaging studies from the beginning; early work developing XFM instrumentation already involved images of diatoms.^267^ The first elemental distribution maps of eukaryotic algae using fully developed XFM setups were also taken from diatoms, *T. weissflogii*, and natural isolates with 0.5 μm step size at the Advanced Photon Source (APS, Argonne, USA).^268,269^ The authors established the quantitative capabilities for Si, Mn, Fe, Ni, and Zn in single cell analysis and determined the detection limits of the technique. The setup was later used by Twining *et al.* to determine the Fe distribution in natural diatom and dinoflagellate isolates upon ocean iron fertilization,^270^ or in specific oceanic regions,^271^ Adams *et al.* used it to determine Cu distribution in the diatoms *Phaeodactylum tricornutum* and *Ceratoneis closterium*, as well as in the green alga *Tetraselmis* sp.^272^ Nuester *et al.* determined the Fe distribution in a similar setup with improved resolution, 0.2 μm step size, in the diatoms *T. pseudonana* and *T. weissflogii.*^273^ Another diatom, *Cyclotella meneghiniania*, was used in a study by de Jonge *et al.*, significantly improving the resolution of XFM tomography to a useful range (<400 nm) for single cells smaller than 10 μm diameter.^274^ Diaz *et al.* demonstrated the utility of XFM for green alga research, using Chlamydomonas and Chlorella species to demonstrate Fe sequestration in stationary cells.^275^ Elemental maps of Fe, Zn, and K were obtained from frozen hydrated Chlamydomonas cells with <100 nm spatial resolution during the development of the Bionanoprobe at the Advanced Photon Source.^223^ The alga, together with a different green alga, *Ostreococcus* sp., was also the subject for demonstrating the utility of ptychography, a coherent diffraction imaging technique that uses multiple overlapping regions of a cell to provide superior spatial resolution. Deng *et al.* demonstrated <20 nm spatial resolution of ultrastructure imaging of frozen-hydrated algae with ptychography while simultaneously recording fluorescence spectra to determine the intracellular P, Ca, K, and S distributions.^193,276^ 3D reconstruction of cellular P, Ca, S, Cl, and K distributions and ultrastructure from ptychographic tomography using GENFIRE also took advantage of Chlamydomonas.^277^ Outside of method development, XFM has proven useful for the characterization of intracellular metal sequestration sites in Chlamydomonas at the APS, and at the European Synchrotron Radiation Facility (ESRF, Grenoble, France). Researchers found Fe, Cu, and Mn to be sequestered in cytosolic vacuoles, acidocalcisomes,^184,194,195^ while the heavy metal Cd was found to be localized both in acidocalcisomes and the pyrenoid in the chloroplast.^196,201^ Similarly, the green alga *Coccomyxa actinabiotis* was analyzed at 100 nm resolution in studies aimed at identifying the mechanisms for Co and Ag tolerance.^278^ Coccolithophores, eukaryotic algae from the haptophytes and renowned for their calcite exoskeleton, were analyzed for elemental distributions up to Sr using XFM at 50 nm resolution in artificial seawater enriched with trace nutrients.^279^ They were also the subject of an X-ray ptychography tomography study at 30 nm resolution determining their ultrastructure.^280^

## Controlling variance in elemental composition of algae

Successful elemental imaging for elemental imaging starts with a controlled elemental environment during cultivation of the alga. For this reason, a chemically defined medium is superior to a complex medium recipe. For Chlamydomonas, the most popular media are TAP/TP and HS/HSM (± acetate as a reduced carbon source), which are both chemically defined.^281^ Avoiding components like sea water, peptone, or yeast extract would be ideal if possible. Both Chlamydomonas media originally used a trace element mixture recipe developed by Hutner,^282^ which can vary substantially in its content in between batches. Hutner’s solution was not specifically optimized for the alga; specifically, it lacks Se completely but contains both Co and B, which are not utilized by the alga.^283^ Instead of the single trace element additive derived from Hutner, a 7-solution trace element suite was developed specifically for *C. reinhardtii*, which changed the composition accordingly and additionally adjusted the concentrations of the other trace elements (especially Zn but also Fe, Cu, and Mn) to better match the algae's metabolic demand.^283^ The use of controlled, high-purity chemicals for media preparation additionally ensures reproducible conditions. The water, glass, and plasticware used for media preparation and cultivation should be low in contaminants. Acid-washing of glassware is recommended, overnight incubation in 6N HCl followed by thorough rinsing in clean water to remove residual HCl.^284^ Depending on the condition of interest, liquid pre-cultures used for inoculation at a specific cell density, already grown in the elemental condition intended for analysis, additionally improve the reproducibility between experiments, which can be useful if there are long periods in between replicates (e.g. in between beamline visits).

## Sample preparation for XFM measurements

The most crucial component of sample preparation is the trade-off between the necessity to deliver the sample material in the required form to the imaging application of choice and to simultaneously preserve the sample material in an unaltered state that reflects the condition that is analyzed.^217^ Long incubation steps, changes in temperature, necessary concentration steps (centrifugation), or buffer changes to accommodate fixation can potentially alter the state of the sample material on its way from its habitat (or the growth chamber in a laboratory) to the imaging application. For photosynthetic organisms, changes in illumination and aeration during sample preparation are also potentially critical, as they can affect metabolism quickly.^285,286^ Metal-sensitive probes compatible with live cell imaging are probably the gold standard in this regard, allowing the researcher to keep cells as close to the state of interest as possible. For XFM, rapid vitrification of cells in liquid ethane (e.g. using a FEI Vitrobot Mark IV plunge freezer^193,223,276,277^ or similar) is minimally invasive, and preserves the cell in a frozen hydrated state. Successful settings for vitrification of Chlamydomonas on the Vitrobot were reported at a temperature between 20 and 22°C, humidity of 100%, with a blot time of 2 s at blot force 0, blot total 1,^193,276,277^ or a blot time of 3 s at blot force 2, blot total 1, wait and drain time of 0 s.^195^ For this to be effective, the beamline needs to support imaging at low temperatures,^223,226^ and the samples to never thaw after the initial freezing event. A major limitation to using vitrification for sample preparation is the availability of cryo‐XFM instruments capable of performing subcellular analyses at low temperatures, and access to specific equipment like plunge freezers can also be prohibitive. Additionally, cryo-preserved samples can collect ice deposits from environmental humidity, especially during, even very brief, transfers between any type of equipment (plungers, microscopes, XFM instruments, and storage/shipping vessels), which can result in a loss of the ability to produce a useful XFM image from the sample. Alternatively, chemical fixation (e.g. using 4% paraformaldehyde) at an early stage of sample preparation is still used and effective, and allows imaging to take place at XFM instruments at room temperature. The elements of interest are crucial in the choice of fixation for any specific study. Chemical fixation, especially using various popular aldehydes, can be problematic for the preservation of the native state, especially of highly diffusible ions, e.g. Na, K, and Cl.^226,287^ A previous study^195^ showed that the intracellular amounts of Fe and Cu were similar in vitrified and chemically fixed cells, and the distribution patterns were comparable with chemical fixation, albeit not as crisp as in the frozen-hydrated cells. Aldehydes can also alter membrane permeability,^287^ which might affect intracellular distribution.

In both cases, the cell material needs to be transferred either to film^196^ or Si_3_N_4_ windows,^193–195,223,276,277^ compatible with the downstream beamlines, prior to freeze-plunging or chemical fixation. More elaborate analyses like ptychography or tomography reconstruction might require specific sample holders or additional sample preparation, which should ideally already be considered at this stage. The Si_3_N_4_ windows can be pretreated with poly-L-lysine to improve adhesion of alga cells (a single droplet of poly-L-lysine applied to the window and incubated for 30 min at 37 °C before the remainder of the droplet is removed and the cells are spotted). For quantitative applications, the cells need to be freed from media remnants,^195,196^ with brief washing steps (ideally with water as the last step), which has to be done prior to freeze plunging,^278^ but can be done after chemical fixation, when cells are already spotted on the carrier.^195^ Washing is crucial, but extended washing can reduce the number of cells on the sample holder. The force by which the washing solution is applied to and removed from the sample holder contributes to the displacement. Using poly-L-lysine to assist in adhesion, a good starting point for the cell concentration of a motile Chlamydomonas culture to be spotted on a Si_3_N_4_ window was found to be 1 × 10^7^ cells/ml, spotting between 50 and 100 μl.^195^ The concentration of the cells on the sample holder is crucial and should be optimized with the sample preparation procedure in place at a light microscope beforehand. If too many cells are spotted, then the analysis becomes significantly more complex, as X-rays can penetrate multiple layers of cells, and the resulting fluorescence will be reflective of all cellular material in the path of the beam. The identification of individual cells at the beamline becomes also more tedious, curiously, too little cells on the holder will do the same, by increasing the time of search and movements in between the positions of individual cells; a healthy balance is therefore ideal. In addition, the emitted fluorescent X-rays used to identify elemental speciation at each spot penetrate biological material less efficiently as the incident X-ray beam, which might result in a loss of fluorescent signal in thicker samples, affecting either the accuracy of the quantitation or even the correct identification of the elemental content at each spot. Controls, like a comparative quantitative analysis of bulk material in parallel to the imaging analysis, allow one to identify problems of this kind during method development.^195,288^

## Determining elemental distribution maps with XFM

Measurement at the individual beamlines will be dominated by the requirements and specifications of the respective instruments. Samples should be in a state, either by fixation or freezing, to withstand the loading and analysis procedures. Calibration of the X-ray fluorescence emission data is required to allow absolute quantitation of the spatially distributed data. The principle and different strategies are nicely summarized in this review.^289^ Briefly, calibration is usually done by comparing the intensity of the fluorescence signal emitted from the individual samples to a calibration curve developed from the measurement of standard thin films containing a few elements with known concentrations.^290^ Matrix matching of the standards and samples is very difficult,^289^ standards used can therefore be quite different in nature from the biological material analyzed. An in-parallel analysis of the elemental composition of the bulk sample material provided for the spatial analysis by other means, e.g. ICP-MS, is therefore of advantage and can be used at later stages to interrogate the spatial quantitation of the material.^195,278^ Additionally, single-cell organisms like Chlamydomonas grown in asynchronous conditions in constant light (the most typical way) will contain a mixture of cells at various stages in the cell cycle.^291–294^ The elemental composition of cells can vary with the growth stage of the cells, and specific observations with respect to the elemental composition might be tied to an individual stage of the cell cycle. Synchrotron beamline time allocation is a crucial limiting factor, especially for quantitative experiments, with respect to the number of cells that can be analyzed at the required spatial resolution. Nevertheless, similar to other microscopy techniques, a mixture of cells from different developmental stages should be selected, as much as possible, to reduce the risk of conclusions based on a biased subset. The size of a cell can inform ‘on which developmental stage’ an individual cell in a batch culture from an asynchronous population currently resides; therefore, selecting cells of various sizes is a straightforward way to avoid oversampling of a specific condition. This can either be done prior to beamline visits, using a light microscope and some coordinate system to record the cell position, or at the beamline. Scans at a lower resolution prior to data collection are routinely used to minimize instrument time by optimizing the focus area of the cell of interest. They also allow for a first assessment of cell size and ensure cell integrity prior to analysis. This selection process, while crucial, can later contribute to the variance in the quantitative data, especially when the data are normalized per cell.

## Data analysis of spatially distributed elemental data

Analysis of the acquired emission spectra can be divided into two parts: an accurate quantitation of the data coming from the instrument and a subsequent analysis of the data in between different cells and conditions using normalization and statistics. In the first part, different options and strategies are discussed in detail in an excellent review.^221^ In brief, quantification of fluorescence data can already be achieved by simple binning of counts within a certain energy window. While this can be sufficiently accurate, peak-fitting to determine the area in the same energy window, especially when combined with correcting for background levels or overlapping peaks, has been demonstrated to be more accurate and should be preferentially used if available. Software tools^230,295–297^ greatly facilitate all aspects of image analysis, including fitting, ROI analyses, and co-localizations. A detailed step-by-step protocol on how to perform and evaluate XFM data fitting using the MAPS software package can be found here.^298^

For the subsequent quantitative image analysis, software tools are also of great assistance because they allow the extraction of the quantified information for each pixel and can summarize it both for the whole image globally and for selected regions of interest. Initially, we recommend dividing the image into two regions: the part of the image that is covered by the cell and the surrounding region.^195,278^ This can be done either manually or algorithm assisted using an abundant element with good signal-to-noise contrast between the cell and the background, e.g. S. Both regions contain valuable information. The background, within the same image, contains the signal recovered from the sample holder and remnants of the sample preparation process, which should be subtracted from the biological material to obtain the total cellular content for each of the identified elements. XFM results are determined as the amount of element/area, summarizing the total amount of material in the column of the beam. For background correction, the amount/area in the background is subtracted from the amount/area in the area covered by the cell before the total cellular amount is calculated from the area covered by the cell. The amounts of elements in the background region should ideally be small compared to the cell image; if there is a substantial amount of elements of interest in the background, then additional washing steps should be added to the sample preparation procedure for the next batch; two consecutive washes of the already spotted cells on the sample holder with clean water were sufficient to have <1% of elements of interest/area in the background region compared to the region covered by the cell. The background-corrected cellular content obtained this way can directly be compared to alternative means of quantitation (e.g. ICP-MS). The quantitative data from this analysis can also be used to evaluate technical aspects, like different approaches to fixation (chemically fixed versus frozen hydrated samples from the same batch), in-between batch variation, or different sample preparation strategies.

**Fig. 3:**
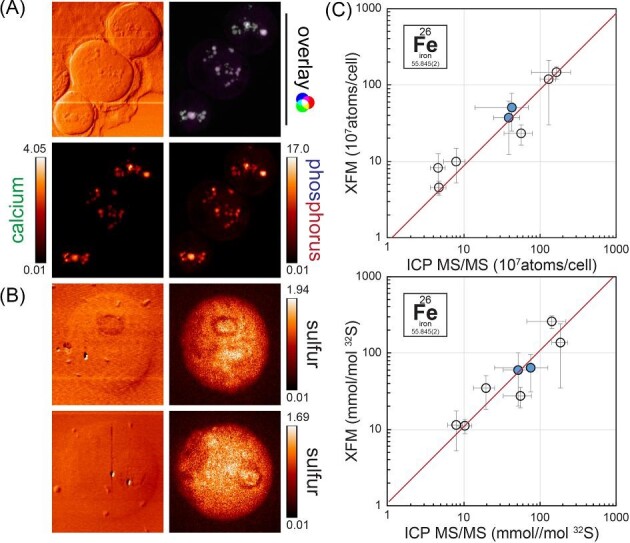
Element distribution identifying organelles and spatial normalization using S. (A) Phosphorus and calcium distribution in Chlamydomonas cells, as well as the overlay, showing acidocalcisomes in Zn deficiency. (B) Sulfur distribution in Chlamydomonas cells, potentially showing starch sheets and pyrenoids. (C) Correlation of Fe content as measured by X-ray fluorescence microscopy (*x*-axis) and ICP-MS/MS (*y*-axis), normalized either per cell (top) or using S content (bottom), also determined either with ICP-MS or via XFM. Error bars in *x* and *y* directions indicate standard deviation in the measurements between at least four individual cells (XFM) or between at least between three independent cultures (ICP-MS/MS). Blue fill indicates frozen hydrated samples; gray fill indicates chemical fixation.

Comparisons between different intracellular features are achieved by identifying individual organelles from differential phase contrast images or ptychography reconstruction, or by algorithm-assisted identification of specific elemental signatures within the cells. In Chlamydomonas, acidocalcisomes, cytosolic vacuoles with a role in trace metal sequestration (Fig. 3A), are rich in P and Ca, and potentially also K;^184,194,195,276,299^ contractile vacuoles, involved in osmoregulation, are rich in K and Cl;^254,300,301^ pyrenoids, sites of concentrated CO_2_ fixation in the chloroplast, might have slightly increased S content;^196^ and large starch shields surrounding the pyrenoid might be visible by the absence of individual elements (Fig. 3B). Other organelles or cellular structures can have a distinguishable elemental composition only in specific nutritional or developmental stages or upon genetic changes, which might then also present an opportunity for selection and further analysis. When algorithm-assisted means are used, it is important to ensure that all the identified regions are completely contained within the cell of interest. Remnants from cell lysis or other cells within the same imaging window might complicate the issue. When intracellular regions are compared, additional normalization or statistical evaluations are necessary to account especially for variance in thickness of the material in the way of the beam. Areas in the center of single cells, where thickness is high compared to peripheral regions, will naturally have a higher amount of element in the 2D projection. Several methods to determine thickness experimentally are discussed previously;^221^ a more direct way to deal with bias is to utilize a strength of XFM and identify a different elemental distribution from the same cell that shows a uniform distribution, e.g. S (Fig. 3C), and use that for normalization of enrichment analysis.^195^

## Conclusion

Eukaryotic algae are crucial primary producers in soil, oceanic, and freshwater environments. Their habitats suffer from low trace nutrient bioavailability or heavy metal pollution, sparking research interest into the mechanisms of their metal metabolism. The strategies involved in trace metal distribution can be exploited for biotechnological utilization, either to improve algae growth and consequently their potential for carbon sequestration, for biofortification, or to utilize their mitigation strategies in bioremediation applications. XFM can be a powerful tool to investigate the intracellular elemental distribution of (trace) nutrients in eukaryotic algae quantitatively, allowing to assign function to individual components involved in managing elemental homeostasis or identifying acclimation strategies or useful phenotypes. This Tutorial Review summarizes the state of research involving subcellular elemental distributions determined using XFM with eukaryotic algae as subjects, and provides a workflow of a quantitative elemental distribution analysis for eukaryotic alga.
